# Block Face Scanning Electron Microscopy of Fluorescently Labeled Axons Without Using Near Infra-Red Branding

**DOI:** 10.3389/fnana.2018.00088

**Published:** 2018-11-06

**Authors:** Catherine Maclachlan, Daniela A. Sahlender, Shuichi Hayashi, Zoltán Molnár, Graham Knott

**Affiliations:** ^1^BioEM Facility, School of Life Sciences, École Polytechnique Fédérale de Lausanne (EPFL), Lausanne, Switzerland; ^2^Department of Physiology, Anatomy and Genetics, University of Oxford, Oxford, United Kingdom

**Keywords:** correlative light and electron microscopy (CLEM), axons, scanning electron microscopy, neuron ultrastructure, serial block-face electron microscopy (SBEM)

## Abstract

In this article, we describe the method that allows fluorescently tagged structures such as axons to be targeted for electron microscopy (EM) analysis without the need to convert their labels into electron dense stains, introduce any fiducial marks, or image large volumes at high resolution. We optimally preserve and stain the brain tissue for ultrastructural analysis and use natural landmarks, such as cell bodies and blood vessels, to locate neurites that had been imaged previously using confocal microscopy. The method relies on low and high magnification views taken with the light microscope, after fixation, to capture information of the tissue structure that can later be used to pinpoint the position of structures of interest in serial EM images. The examples shown here are td Tomato expressing cortico-thalamic axons in the posteromedial nucleus of the mouse thalamus, imaged in fixed tissue with confocal microscopy, and subsequently visualized with serial block-face EM (SBEM) and reconstructed into 3D models for analysis.

## Introduction

Scanning electron microscopy (SEM) has become the tool of choice for many investigations of cell and tissue 3D ultrastructure. Serial block-face SEM (SBEM; Denk and Horstmann, 2004), focused ion beam SEM (FIBSEM, Knott et al., 2008), array tomography (Micheva and Smith, 2007) and automated tape collecting ultramicrotome SEM (ATUM-SEM; Schalek et al., 2012) provide biologists with the means to image large volumes of biological material with enough resolution to see all organelles and membranes (reviewed by Briggman and Bock, 2012). The methods have proved particularly useful in neuroscience as significant portions of neural circuits can now be visualized and mapped. However, identifying different types of cells, or parts of cells, with EM is not straightforward. Many different immunocytochemical or tracing approaches can be combined with EM, but these often require the cellular material to be less stringently fixed or permeabilized in such a way as to allow marker molecules, such as antibodies, free access, or quite often both (Knott et al., 2009). This reduces the quality of the ultrastructure and leads to difficulties in interpreting the images. The vast array of molecular biology manipulations that can label molecules, cells and tissues with fluorescent markers offers a myriad of possibilities for light microscopy. Therefore, combining the two by imaging initially with light microscopy, and then subsequently finding the same structure in the serial EM images offers significant advantages. These include the opportunity to combine *in vivo* imaging data from the light microscopy (LM), with the ultrastructural analysis of the same structures that have been optimally preserved. This correlated approach is used frequently in single-cell experiments (Murphy et al., 2011), especially cells cultured as a single monolayer, but in tissue volumes this is more difficult, and various strategies have been used to locate the fluorescently labeled structures in the EM image stacks without using immunocytochemistry. In very small structures, such as the *Drosophila* neuromuscular junction, serial sectioning of the entire muscle is possible and allows single axonal boutons, previously imaged *in vivo*, to be found with EM (Zito et al., 1999). For structures located in much larger pieces of tissue such as the mouse brain, or retina, the vasculature is used to identify the region and this is then completely sectioned and imaged prior to identifying individual neurons of interest afterward (Bock et al., 2011; Briggman et al., 2011). Targeting small structures and reducing the volumes that need to be cut and imaged is possible by laser branding the fixed tissue to create fiducial marks (Bishop et al., 2011). This is a proven technique for localizing neurites that had previously been imaged *in vivo* (Maco et al., 2014). Small (approximately 10–50 micrometer) squares around the structures of interest can be seen in the resin embedded tissue, and in the EM images, giving the opportunity to indicate the position of the structures of interest. This has been used in a number of correlative studies with *in vivo* 2 photon microscopy (Grillo et al., 2013; Mostany et al., 2013; Cane et al., 2014). However, while this is an effective approach, a 2-photon laser system may not always be at hand, particularly when not *in vivo* imaging.

For these reasons, we developed a method, using SBEM, that does not require introducing any fiducial marks, or the need to section and image massive volumes of tissue to reliably find axons and dendrites previously imaged with light microscopy. The approach relies on the natural landmarks, such as blood vessels and cell bodies. It only requires low-resolution imaging, of the entire section, using transmitted light, combined with high-resolution confocal imaging of the structures of interest. Once the tissue section is heavy-metal stained and resin embedded, careful block preparation using the blood vessel pattern and trimmed edges, allows regions of interest to be accurately positioned ready for EM. SBEM imaging can then collect both high and low-resolution images that reveal the exact location of the relevant structures.

The reliability with which SBEM can collect serial images of structures that were previously imaged with light microscopy removes the need to convert fluorescent markers to electron dense stains. This gives opportunities to carry out combined light and EM analyses using a wide range of different types of fluorescence imaging. To demonstrate this method, we show how fluorescent cortico-thalamic axons, and their boutons that synapse with neurons in the posteriormedial thalamic nucleus, can be imaged with laser scanning confocal microscopy and then 3D reconstructed from serial electron micrographs using SBEM. The structure of axons communicating between the thalamus and cortex have been the focus of many ultrastructural studies. These have used a variety of labeling strategies to locate them including tracers such as lectins (Hoogland et al., 1991) or biotinylated dextrans (Li et al., 2003), lesions (Mathers, 1972), autoradiography (Ogren and Hendrickson, 1979) and immunocytochemistry against endogenous markers (Godwin et al., 1996; Groh et al., 2014) or fluorescent tags expressed in axons (Hoerder-Suabedissen et al., 2018a).

## Methods

### Tissue Preparation

The animal experiments were performed in the animal facilities of the University of Oxford (UK) under a valid Animals (Scientific Procedures) Act project license as well as with local ethical approval by the central Committee on Animal Care and Ethical Review (ACER) and the Animal Welfare and Ethical Review Body (AWERB) at the University of Oxford. Adult mice containing a Cre-recombinase expressing strain (Tg(Rbp4-cre)KL100Gsat/Mmucd (Rbp4-Cre; Jackson Laboratories) were crossed with B6;129S6-Gt(ROSA)26Sortm14(CAG-tdTomato)Hze/J (Ai14) to label cortical layer 5 neurons. The axons of these Rbp4-Cre;Ai14 mice were visible in the posterior medial thalamic nucleus (POm; Grant et al., 2016; Hoerder-Suabedissen et al., 2018b). Mice at P18 were perfused with a buffered solution of 2.5% glutaraldehyde (Electron Microscopy Sciences, 16220), and 2% paraformaldehyde (Electron Microscopy Sciences, 15714) at pH of 7.4. The brain was then removed and embedded in agarose, and 80-micrometer thick sections cut with a vibratome in the coronal plane, at the level of the thalamus. Only sections containing the posterior medial nucleus were collected.

### Collecting of Fluorescence and Light Microscopy Images

**Figure 1:**
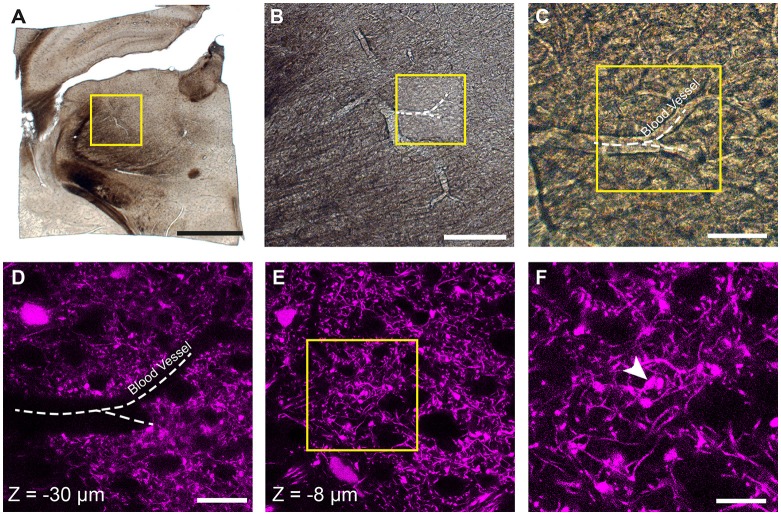
Locating fluorescent structures in an 80-micrometer thick coronal brain section from a P18 Rbp4-Cre;Ai14 mouse. **(A)** Low magnification image of the thalamic region in the vibratome section. The yellow box indicates the region shown in **(B)**. **(B,C)** Higher magnification views of the region containing the fluorescent axons of interest showing a prominent blood vessel indicated with a white dotted line. Yellow box in **(B)** indicates region shown in **(C)**. **(D)** Confocal image of corticothalamic axonal boutons in the region indicated in **(C)**. The dark shadow of the same blood vessel is seen at a depth of 30 micrometers. **(E)** At a shallower depth of 8 microns (*z* = −8 μm) a specific bouton is singled out from imaging with electron microscopy (EM). **(F)** Higher magnification view of the region indicated with the yellow box in **(E)**. White arrowhead indicates a single bouton that is targeted for ultrastructural analysis. Scale bar in **(A)** is 1 mm; **(B)**, 200 μm; **(C)**, 50 μm; **(D)** 25 μm; **(F)**, 10 μm.

Prior to confocal imaging, the sections were viewed under a dissecting microscope and using a scalpel the region of the thalamus was trimmed away from the rest of the brain (Figure 1A). This created a section of approximately 4 × 4 mm. These pieces were then imaged with both bright field and epi-fluorescent illumination to capture the entire section (Figure 1A) and then at increasingly high magnifications so that blood vessels could be seen in each image (Figures 1B,C), and also their proximity to the fluorescent axon terminals of interest recorded (Figures 1D,E). At this point, confocal microscopy was used to capture images of the fluorescent axons (LSM710; Zeiss). Image stacks were collected with 0.5 μm distance between images at using a ×63 objective. These are crucial for locating the fluorescent axons in the final EM image series. In the example shown in Figure 1, the blood vessel is seen at a depth of 30 microns (Figure 1D), however, the axonal bouton of interest lies only 8 micrometers below the surface (Figures 1E,F).

### Preparation of Tissue for Electron Microscopy

The trimmed and imaged sections were then transferred to cacodylate buffer (0.1 M, pH 7.4), and heavy metal stained with a protocol largely similar to that described by Hua et al. (2015). In brief, the sections were postfixed in 1.5% potassium ferrocyanide (Sigma 14459-95-1) and 2% osmium tetroxide mixed together (Electron Microscopy Sciences, 19110), then stained with 1% thiocarbohydrazide (Sigma, 101001342) followed by 2% osmium tetroxide and then further stained overnight in 1% uranyl acetate (Electron Microscopy Sciences, 22400). They were then washed in distilled water at 50°C before being transferred to a lead aspartate solution at pH 5, at the same temperature. After 20 min the sections were rewashed in distilled water at room temperature and then dehydrated in increasing concentrations of ethanol followed by increasing amounts of Durcupan resin (Electron Microscopy Sciences 14040 Parts A, B and D replace C with DMP30 from Electron Microscopy Science 13600) until at 100%. After infiltrating overnight, the sections were placed between glass microscope slides coated in a mold separating agent (Glorex Inspirations, Switzerland; 6 2407 445) and the resin hardened at 65°C for 24 h.

### Preparation of Block Ready for SBEM Imaging

**Figure 2:**
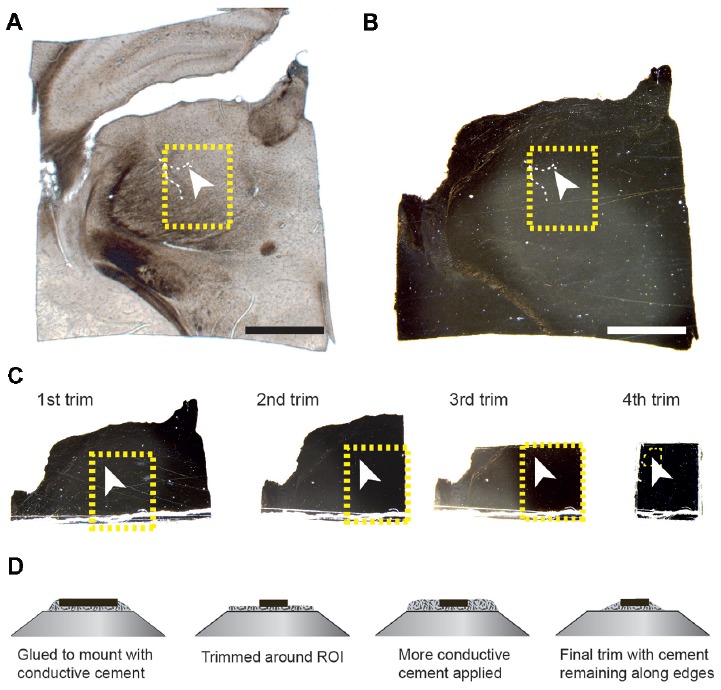
The region of interest can be located in the tissue section using blood vessels and the edge of the tissue section as guides. The resin-embedded tissue is trimmed according to these landmarks. **(A)** The wet section is imaged with transmitted light to show the position of the blood vessel in relation to the entire section. The arrowhead indicates the same highlighted blood vessel shown in Figures 1A–D. The white arrowhead in **(A–C)** indicates the exact same position in the tissue. **(B)** After the section has been heavy-metal stained, and resin embedded, the same region can be localized by overlaying the image of the wet section (shown in **A**) with that of the resin-embedded section, whether or not the blood vessels are still visible. **(C)** This region is trimmed from the rest of the section by sequentially removing one side of a square that contains the region of interest. The yellow boxes in the first three trims indicate the final region stuck to the stub. After each trim, an image is taken and this is overlaid with the previous so that the yellow box can be positioned precisely, indicating the region of interest. The small yellow box shown in the 4th trim shows the final region that remains after trimming in the ultramicrotome. This region corresponds with the block face shown in Figure 3. **(D)** The schematic diagrams show the initial and final trimming of the block from the side that is mounted on the pin. The block is initially trimmed to leave only the region of interest of approximately 250 × 250 μm. This is then surrounded in the conductive glue again, and this is trimmed away again until some of the resin remains around the edge to help with conduction. Scale bar in **(A,B)** is 1 mm.

Once the resin had completely polymerized, the glass slides were removed, leaving a thin resin lamella containing the section (Figure 2B). However, at this point, the section is well impregnated with heavy metals and completely opaque to transmitted light. It is therefore difficult to see any histological features other than bright spots of light from the blood vessel lumens traversing vertically through the section (Figure 2B). To locate the region of interest, images of the entire section are overlaid and aligned with the image of the same section prior to embedding (Figures 2A,B). There is no shrinkage at this stage as little deformation of the second image (Figure 2B) is required to align onto the first (Figure 2A). On this first image of the unstained section, it is possible to pinpoint the region of interest, from the position of the blood vessel, and therefore identify the same region in the stained resin embedded section (highlighted with a yellow box in the Figures 2A,B). These two overlaid images are then used to indicate from where excess material can be trimmed. It is important to note that blood vessels lying horizontally in the section, and visible in the wet section, may not be visible in the resin embedded section. Therefore, the accurate alignment of the two images is important, using the edges, so that the region of interest can be identified. In this example, a large vessel is being used as a fiducial mark for the smaller fluorescent structures. Smaller vessels radiate away from such structures providing a unique pattern throughout the entire section. This allows any regions to be found back in the EM as the closeness of blood vessels means they are never too far from fluorescent structures. An indication of this is given in the analyses of vasculature in different regions of rodent brain (Schlageter et al., 1999). In sections through the cerebral hemispheres the average distances between microvessels are between 17 and 26 micrometers. Although the smallest capillaries may not be clearly visible when focusing through wet, thick sections, in our experience a blood vessel is always seen within approximately 20 microns of a structure.

Using a razor blade, parts of the section are removed (Figure 2C). This is done by first cutting one side, and then recording an image of the remaining piece. This image is then overlaid with the previous one, showing exactly how much material was removed. This process is repeated for the adjacent side, and again the image overlaid with the previous. Repeating this for each of the four sides leaves a small block with the region of interest located in the center (Figure 2C), and importantly, its location in the original section known. Typically, the remaining piece of the section is approximately 1 mm × 1 mm. This can then be glued to the SEM pin (Micro to Nano place, 10-006003-50) using electrically conductive epoxy resin (Ted Pella Inc., Redding, CA, USA 16043), and left to harden completely at 65°C, overnight.

**Figure 3:**
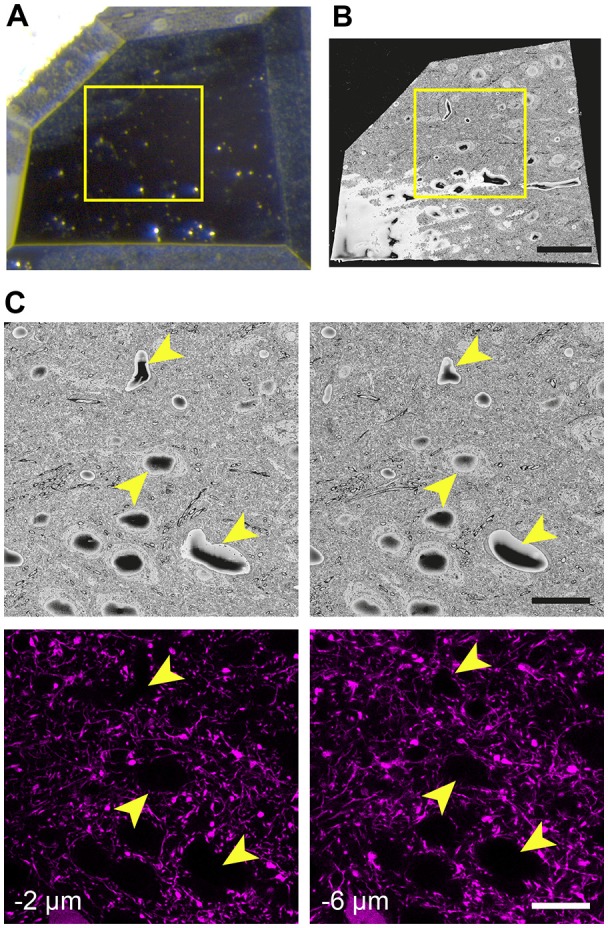
The final trimmed block is scanned in the microscope revealing the position of cell bodies and blood vessels seen with fluorescence microscopy. **(A)** The block is carefully trimmed so that the region of interest lies just within its borders. An image of this final block can be overlaid precisely with an image of the entire region taken prior to trimming. Then, a box (yellow) is able to indicate the precise region where images can be taken. **(B)** The block is then placed in the microscope and once a large part of the face has been revealed with the diamond knife, an scanning EM (SEM) image is taken. **(C)** The region of interest is then confirmed by matching the position of cells and vessels (arrowheads) with those seen previously in the confocal image stacks. Their height in the image stacks is also noted. Scale bar in **(B)** is 50 μm and in (**C**; right-hand images) is 20 μm.

When the glue is completely hardened, the pin is mounted to the arm of an ultramicrotome and with glass knives a block face of approximately 250 μm × 250 μm trimmed. As before, an image is taken after each side is cut away and overlaid with the previous image to indicate the position of the remaining tissue. This allows the region of interest to be positioned close to the center of the block (Figure 3A). To avoid any confusion as to the orientation of the block, a trapezoid shape is cut. It is important to trim down through the entire thickness of the block, leaving at least 80-micron proud of the pin.

This block is then again surrounded with electrically conductive epoxy resin and left to harden at 65°C overnight. The reason for this second application of glue is to maximize the amount of electrical contact between the tissue and the pin. The second glue layer is trimmed away, using a glass knife in the ultramicrotome, being careful not to remove more resin from the block, and not too deep as was previously trimmed (see schematic diagram in Figure 2D). This means that the resin embedded tissue is now surrounded by the conductive resin, which will help to disperse the charge when the sample is imaged. Any excess resin is then removed from the surface of the block until the surface of the tissue is exposed. At this point, the block is ready and is gold sputter coated with a 50 nm layer (Quorum Technologies; Q300T).

### SBEM Imaging

The sample is loaded into the SEM microscope (Merlin, Zeiss NTS) fitted with the 3View cutting system (Gatan, Inc., Pleasanton, CA, USA). While the microscope chamber is open, the initial cutting of the face is performed to ensure that the cutting window is in the correct position, the block is seated securely, and sections are cleanly removed. The door is closed and the microscope pumped down to high vacuum ready for imaging.

**Figure 4:**
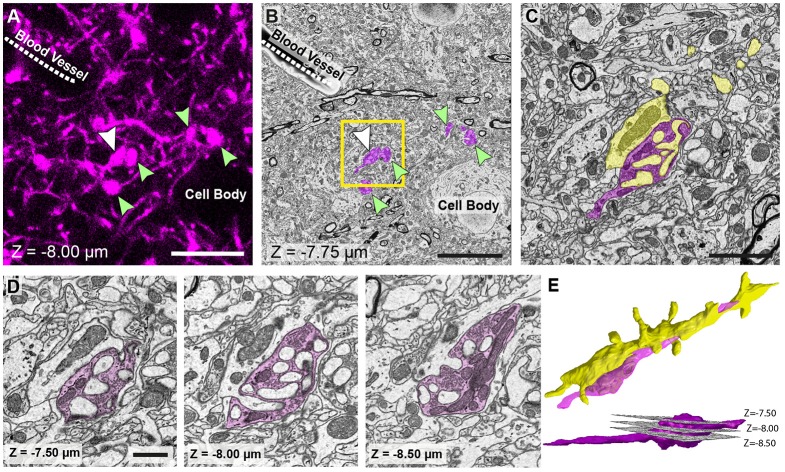
The matching of structures seen in both fluorescence and EM images allows features of interest to be identified and serially imaged. **(A)** Fluorescent image of labeled axons and their boutons (arrowed) shows their location in relation to the shadows of a cell body and blood vessel (labeled). Bouton indicated with a white arrow is the same bouton from Figure 1. **(B)** EM micrograph of the same region shown in **(A)** with the corresponding features labeled. These features are used to pinpoint the position of the axons and axon terminals (pseudo-colored in purple). **(C)** Higher magnification view of the highlighted region in **(B)**. **(D)** The axonal bouton shown in three images from the series, and from which the reconstruction is made. The depth of each image in the stack is shown at the bottom left of each image. **(E)** Reconstruction of the bouton (purple) shown in **(D)** and the dendrite (yellow) to which it synapses. Lower image, side view shows the position of each of the images shown in **(D)**. Scale bar in (**A,B**; left hand image) is 10 μm; in **(C)** is 2.5 μm; in **(D)** is 1 μm.

The first SEM image is then taken to see the entire block face. This image can be overlaid with all previous aligned images taken during the preparation (Figures 3A,B). In this way, the EM image can be positioned on the LM image of the section prior to any trimming revealing the location of the region of interest on the block face. After this alignment, the imaging window can be positioned on the block face. However, it is important to now look at the serial confocal images to understand which features will be visible in the EM. The most obvious ones are blood vessels and cell bodies which appear dark in the confocal stack (Figure 3C). Their depth in the block can also be estimated from this stack. This Z-depth value is important for indicating when the ROI will appear in the serial EM images. To reach the correct position more quickly, thicker sections can be cut (e.g., 200 nm) while imaging at lower magnification (1K× Magnification, 2 kV, 150 pa and 19 nm resolution) using the backscatter detector. As this approach is carried out it is important to compare each low magnification EM image with the different images in the confocal stack to make sure that the imaging is proceeding as expected and to better pinpoint where in the block the ROI will appear. When the block surface is estimated to be close to the structure of interest (2 μm in the case shown in Figure 3), the higher resolution images can be collected. For this the parameters are: 6000 × 6000—pixel images, 6.5 nm x and y resolution, 2 kV beam tension, 150 pA) and sectioning thickness of 50 nm. This gives a field of view of 39 μm (Figure 4B). The imaging then continues until the entire thickness containing the relevant structures have been imaged.

### Image Processing, Analysis and 3D Reconstruction

The final image series is aligned using the alignment functions in the TrakEM2 plugin of FIJI (Cardona et al., 2012)^1^. Segmentations are then made on suspected structures corresponding to those imaged with LM. These first drawings are done rapidly only to confirm the correct identities by checking their shape with those of the fluorescent structures in the confocal stack. As well as these features, blood vessels and cell bodies are also roughly segmented to orientate the different features in the two image stacks (Figure 4). When it is clear that the correct structures have been found, the same software is used to make the final reconstruction. The models are then exported in the OBJ file format that can be imported as meshes into the Blender software^2^. Within this software, any parts of the meshes can be manipulated and measured, using the NeuroMorph tools (Jorstad et al., 2015, 2018). This includes surface areas, volumes, and distances, as well as tools to see the original EM images combined with the final models so that other features can be added, or their identities checked.

## Results and Discussion

This article demonstrates that fluorescent structures seen with confocal microscopy can subsequently be imaged with EM without using any additional specific markers, introducing any fiducial marks or collecting large EM stacks of entire samples to locate them. Our approach uses fixatives that ensure optimal preservation of the ultrastructure, i.e., glutaraldehyde. In the example shown here, the fixation attenuated the fluorescence of the tdTomato deeper into the tissue, but we were still able to collect reasonable images at a depth of 30 μm (Figure 1D). Imaging beyond 40 μm would have been very limited, but as the section was 80 μm thick it would have been possible to flip the section if features of interest were closer to the opposite surface.

We reconstructed axon terminal of layer 5 pyramidal neurons (Rbp4-Cre; Ai14) in PO in this study. The density of these axons in this region is reasonably high, but nevertheless, the shape of individual boutons could be easily distinguished from one another. The advantage of such a labeling is that cell body and blood vessels appear as unlabeled holes in the tissue, and easily seen (Figure 4A). Denser labeling would leave these structures even more evident but would make it difficult to isolate the individual axons. Sparser labeling would limit the ability to see the cell bodies and vessels, but in such a case a fluorescent stain such as DAPI could be used to highlight all the cell nuclei. This would not compromise the quality.

The results show how the imaging capacity of the SBEM is well suited to this correlative light and EM method. The ability to rapidly capture an image of the entire block face that approximately matches the field of view of the LM image (Figure 3C) makes it easy to correlate the different features of the tissue. As soon as the same structures, such as cell bodies, have been identified, the field of view in the SBEM can be narrowed, and the resolution increased, to the region with the structures of interest (Figures 4A,B). For this reason, the method is not as suitable for FIBSEM. Accurately milling such a large field of view, in just a few minutes, would be impossible with an ion beam. In addition, with FIBSEM the region of interest needs to be close (within 20 μm) to the edge of the milled surface so that milling aberrations, such as curtaining, do not occur. Therefore, a much smaller block would need to be prepared with the ultramicrotome, and the risk of removing important distinguishing features would be high. The advantages of the FIBSEM imaging are that lower amounts of heavy metals can be used to stain the tissue to produce images with high contrast. This means that the laser marks can be seen with transmitted light microscopy, even after staining and resin embedding. In the staining protocols that are used for SBEM imaging, the transmitted light is not able to traverse the section, so any laser marks are obscured. This means that the laser marks cannot be used to accurately guide the block trimming process.

The SBEM technique is also able to remove far larger volumes of resin from the block face than the FIB within only a few hours. This is a clear advantage if the region of interest lies deep inside the section. Accurately milling a window of the size shown in Figure 4 would not be possible with FIBSEM.

Therefore, while the resolution of the FIBSEM is superior in the Z plane, resulting in image stacks with near isotropic voxels, it is limited in the size of images that are possible in the x and y plane. This means that collecting images of a similar size to those of the high-resolution light microscopy is impossible, making it hard to match the same feature seen in both modalities: LM and EM.

In summary, for many years neuroscientists have used EM to analyze the connectivity in the brain. With a range of labeling approaches, different types of cells and features can be specifically targeted for ultrastructural analysis. Cell-type specific Cre-recombinase expression enabled us to monitor selective populations of neurons using stop-floxed fluorophores. However, without using any labeling methods that may disrupt the tissue ultrastructure we instead use a correlative approach using landmarks within the tissue to locate precise regions in the EM images and find specific structures seen with light microscopy. By relying only on the light microscopy to provide details of the cell identities, rather than specific staining at the EM level, we show here how the ultrastructure can be maintained and structures such as axons easily targeted and serially imaged at the EM level to allow for 3D analysis, revealing uncompromised details of their morphology, and also that of their synaptic partners.

## Author Contributions

CM, DS and GK conceived the experimental method. CM and SH carried out the experiment. CM, DS, SH, ZM and GK wrote the manuscript. ZM and GK helped supervise the project.

## Conflict of Interest Statement

The authors declare that the research was conducted in the absence of any commercial or financial relationships that could be construed as a potential conflict of interest.

## Footnotes

^1^http://fiji.sc

^2^www.blender.org
